# Manufacture of Highly Transparent and Hazy Cellulose Nanofibril Films via Coating TEMPO-Oxidized Wood Fibers

**DOI:** 10.3390/nano9010107

**Published:** 2019-01-16

**Authors:** Weisheng Yang, Liang Jiao, Wei Liu, Hongqi Dai

**Affiliations:** 1 Jiangsu Co-Innovation Center for Efficient Processing and Utilization of Forestry Resources, Nanjing Forestry University, Nanjing 210037, Jiangsu, China; 15850519171@163.com (W.Y.); ljiaonjfu@outlook.com (L.J.); 2 School of Chemical & Biomolecular Engineering and Renewable Bioproducts Institute, Georgia Institute of Technology, Atlanta, GA 30318, USA; weiliu_toya@yahoo.com

**Keywords:** surface morphology, TEMPO-oxidized fibers, optical properties, hazy

## Abstract

Traditionally, inorganic nanoparticles (SiO_2_, TiO_2_) have been utilized to tune the optical haze of optoelectronic devices. However, restricted to complex and costly processes for incorporating these nanoparticles, a simple and low-cost approach becomes particularly important. In this work, a simple, effective, and low-cost method was proposed to improve optical haze of transparent cellulose nanofibril films by directly depositing micro-sized 2,2,6,6-tetramethylpiperidine-1-oxyl (TEMPO)-oxidized wood fibers (“coating” method). The obtained films had a high total transmittance of 85% and a high haze of 62%. The film samples also showed a high tensile strength of 80 MPa and excellent thermal stability. Dual sides of the obtained films had different microstructures: one side was extremely smooth (root-mean-square roughness of 6.25 nm), and the other was extremely rough (root-mean-square roughness of 918 nm). As a reference, micro-sized TEMPO-oxidized wood fibers and cellulose nanofibrils were mixed to form a transparent and hazy film (“blending” method). These results show that hazy transparent films prepared using the “coating” method exhibit superior application performances than films prepared using the “blending” method.

## 1. Introduction

Tuning optical performances including transparency and optical haze play a critical role in preparing optoelectronic devices with varying optical requirements. For outdoor display [1] or solar cells [2,3], high transmittance and high optical haze are essential. Optical haze is defined as the ability to scatter incident light, presenting as translucent or opaque. In general, methods for preparing hazy transparent films can be classified into two types: the particle-diffusing type, which relies on the transparent particles inside the transparent films to scatter light, and the surface-relief type, which relies on microstructures on the surface of the transparent films to scatter light [4,5]. For the particle-diffusing type, transparent SiO_2_ and TiO_2_ nanoparticles and silver nanowires have been utilized for hazy transparent film fabrication due to the difference in the refractive coefficient between transparent substrates and inorganic nanoparticles [6,7]. For the surface-relief type, polydimethylsiloxane (PDMS) replica molding [8], the silver halide sensitized gelatin method [9], holographic recording [10], the 3D diffuser lithograph [11], photofabrication [12], hot embossing [13], and roller extrusion [4] have been developed to replicate the microstructures onto the surface of plastic films. However, most methods employ complex processes and require expensive equipment.

As a side effect of the incorporation cost, novel “green” methods have attracted much attention in recent years. Recently, transparent papers with high haze have attracted abundant attention as a potential bulk substrate for fabricating flexible transparent devices. Flexible and transparent films made of cellulose fibers or cellulose nanofibers are emerging as a substrate in optical electronic devices. Fang et al. (2013) reported a low-cost method for the preparation of a transparent substrate with better shape stability via filling cellulose nanofibrils (CNF) into opaque mesoporous wood fiber-based paper [14]. Zhu et al. (2015) achieved >90% total transmittance and >90% light scattering when the microfibers were in situ, nanowelded in ionic liquid [15]. An effectively light coupling and antiglare effect was demonstrated for the application of Organic Light Emitting Diode (OLED) lighting. Nogi et al. (2017) introduced a hazy transparent nanopaper consisting of cellulose nanofibers and some microsized cellulose fibers with a total transmittance of 89.3–91.5% and haze values of 4.9–11.7% [16]. In these methods, the highly optical haze was mainly achieved through the serious light scattering of the micro-sized fibers within the transparent films, which is similar to particle-diffusing type. Herein, the surface-relief type was presented to fabricate microstructures onto 2,2,6,6-tetramethylpiperidine-1-oxyl (TEMPO)-oxidized cellulose nanofibril (TOCNs) film surfaces to achieve high optical haze. Micro-sized TEMPO-oxidized wood fibers were utilized to coat on TOCN film surfaces to achieve surface-relief microstructures.

In the present study, a highly transparent and hazy TOCN film was fabricated by a simple coating of micro-sized fibers. Dual sides of the hazy transparent films had different microstructures: one side was smooth and the other was rough. The serious light scattering induced by the rough surface endows a high haze for transparent TOCN films. The extremely smooth surface with root-mean-square (RMS) roughness of 6.25 nm is suitable for continuous conducting thin layer depositing. As a reference, micro-sized TEMPO-oxidized wood fibers and cellulose nanofibrils were mixed to form a hazy transparent film (“blending” method). The influence of preparation methods on the transmission haze and surface morphology was investigated.

## 2. Materials and Methods 

### 2.1. Materials

Softwood bleached kraft pulp was used as the original cellulose, purchased from Suzanno. TEMPO-oxidized wood fibers (TOWFs) and TEMPO-oxidized cellulose nanofibrils (TOCNs) were prepared according to previous reports [17,18]. According to previous measurements, the size of TOWFs was around 63.0 μm length and 18.6 μm width. The TOCNs used in this study had a surface carboxylate content of 1.633 mmol/g with a diameter of 6–8 nm and a length around 500 nm.

### 2.2. Fabrication of Hazy Transparent Films via “Blending” Method

The mixture of TOCNs/TOWFs with the mass ratio of 10/3 was prepared from a TOCNs suspension (0.5 wt %) and a TOWFs suspension (0.5 wt %). The obtained mixture was carefully mixed to ensure good dispersion and to prepare hybrid films via the solution-casting method. The solution-casting method was conducted by pouring the mixture slurry into plastic Petri dishes (diameter 60 mm) and dried at room temperature for a few days until a dried film formed. The obtained dried films were denoted as TOCNs/TOWFs-B films with the basis weight of 65 g/cm^2^.

### 2.3. Fabrication of Hazy Transparent Films via “Coating” Method

The TOCNs suspension was diluted to approximately 0.5 wt % and stirred at room temperature overnight to guarantee the uniform dispersion. Then, the homogeneous 0.5 wt % TOCNs aqueous suspension in a plastic petri dish (diameter 60 mm) was dried until a thin TOCNs gel formed. Continuously, the TOWFs suspension with 0.5 wt % was directly coated onto the TOCNs gel and dried under room temperature until a dried film formed. The obtained hazy transparent films were denoted as TOCNs/TOWFs-C films with the basis weight of 65 g/cm^2^. The mass ration of the TOCNs/TOWFs was still 10/3 to ensure comparability. 

### 2.4. Analysis

#### 2.4.1. Surface and Cross-Sectional Morphology 

Surface and cross-sectional morphology were analyzed by field emission scanning electron microscope (FE-SEM, JSM-7600F, JEOL Ltd., Tokyo, Japan) and atomic force microscopy (AFM, Dimension Edge, Bruker, Hamburg, Germany). For FE-SEM characterization, film samples were mounted on the well-polished metal stub and then coated with gold, using Agar HR sputter coater. For AFM measurements, film samples were placed on the mica plate. Images were acquired in tapping mode in air using a silicon cantilever, which was in vibrating mode at 160–225 kHz. 

#### 2.4.2. Optical Properties 

The light transmittance and optical haze were measured using a Ultraviolet/Visible/Near infrared (UV/VIS/NIR) spectrophotometer (Lambda 950, PerkinElmer, Waltham, MA, USA) at a wavelength range from 400 nm to 1100 nm according to the ASTM1003-13 standard method [19].

#### 2.4.3. Thermal Properties

Thermal properties of film samples were analyzed using a thermos-gravimetric analyzer (TGA) (Q5000IR, TA Instruments, New Castle, DE, USA). About 5 mg was heated from 35 °C to 600 °C with a heating rate of 10 °C/min under an inert atmosphere of nitrogen at a gas flow of 20 mL/min.

Thermal stabilities of the film samples under air atmosphere were tested in a muffle furnace. The samples were heated from room temperature to 600 °C while the morphology of the film samples was observed.

#### 2.4.4. Mechanical Properties

Mechanical analysis of film samples was carried out using a tensile tester (H25KT, Tinius Olsen, Horsham, PA, USA) with a 500 N load cell at 1 mm/min at room temperature, according to ASTM D638-03. The samples had a typical dimension of 5 mm in width and 40 mm in length. Prior to the test, all samples were pre-conditioned at 50% relative humidity (RH), 23 °C for at least 24 h. Three measurements were carried out for each sample.

#### 2.4.5. Resistance to Corrosive Chemicals Property

The chemical stability of film samples in diluted hydrochloric acid (0.1 mol/L, pH = 1.0) was estimated by investigating the mechanical properties of film samples before and after treatment. The samples had a typical dimension of 5 mm in width and 40 mm in length.

#### 2.4.6. Resistance to Photodegradation Property

The resistance to photodegradation of film samples under UV radiation was estimated by investigating the mechanical properties of film samples before and after treatment. The samples had a typical dimension of 5 mm in width and 40 mm in length.

## 3. Results and Discussion

### 3.1. Optical Properties

**Figure 1:**
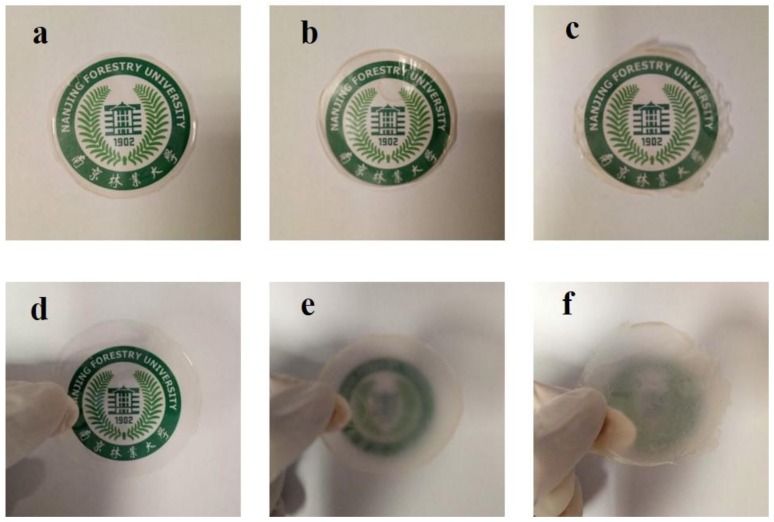
(**a**–**c**) Visual appearance of 2,2,6,6-tetramethylpiperidine-1-oxyl (TEMPO)-oxidized cellulose nanofibril (TOCN) films, TOCNs/ TEMPO-oxidized wood fibers B (TOWFs-B), and TOCNs/TOWFs-C films in close contact with underneath color pattern. (**d**–**f**) Digital photographs of TOCN films, TOCNs/TOWFs-B films, and TOCNs/TOWFs-C films were lifted up to 6 cm above color pattern.

**Figure 2:**
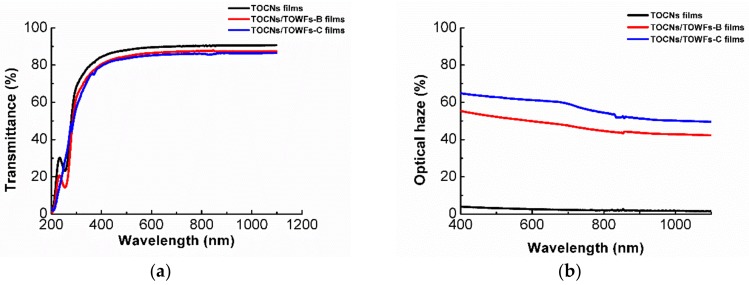
(**a**) Transmittance of TOCN films, TOCNs/TOWFs-B films, and TOCNs/TOWFs-C films and (**b**) Optical haze of TOCN films, TOCNs/TOWFs-B films, and TOCNs/TOWFs-C films.

In comparison with micro-sized fibers, TOCNs with nano-sized dimensions have extremely low scattering for the visible light. The reason for this phenomenon is that the dimension of TOCNs (6–8 nm) is much smaller than the wavelength of the visible light (390–760 nm). The compact structure and low porosity weakens the light scattering between fibers and air, endowing high transparency for the TOCN films [20]. TOCN films, as a bulk optical material for optoelectronics, have attracted abundant attention due to their excellent optical transmittance [21]. However, their low haze is not suitable for preparing some optoelectronics such as outdoor display and solar cells, which need high optical haze. Herein, micro-sized TOWFs with strong light scattering were used to improve optical haze of the TOCN films via the “blending” method or “coating” method. The visual appearance of TOCN films, TOCNs/TOWFs-B films, and TOCNs/TOWFs-C films in close contact with a color pattern is shown in Figure 1a–c, respectively. It can be seen that all the films had high transparency, and the pattern under the transparent films can be clearly observed by human eyes. These results indicated that the existence of TOWFs has a weak influence on the transparency of TOCNs/TOWFs hybrid films. As shown in Figure 2a, all film samples exhibited high transmittance, ranging from 83–89%.

Transparency is a significant optical parameter for most transparent electronic devices [22]. However, for some specific devices such as indoor and outdoor displays and solar cells, optical haze is also an important indicator that cannot be ignored. As the Figure 1d–f shows, the pattern behind the TOCN films was clearly observed, while for TOCNs/TOWFs-B and TOCNs/TOWFs-C films, the bottom patterns look obscure. According to the optical haze data (Figure 2b), the neat TOCN films had an extremely low optical haze of 3.8% at 600 nm. After incorporating TOWFs, the composite films exhibited a high optical haze of 50% for TOCNs/TOWFs-B and 62% for TOCNs/TOWFs-C films. This may be due to TOWFs having prominent light scattering, which had been confirmed by previous studies [23]. Simultaneously, TOCNs/TOWFs composite films had a rougher surface than neat TOCN films. The rough surface caused substantial light scattering, which led to a high transmission haze [24,25]. In comparison to the “blending” method, the hazy film prepared by the “coating” method exhibited higher optical haze. The higher transmission haze could be ascribed to higher surface roughness.

**Figure 3:**
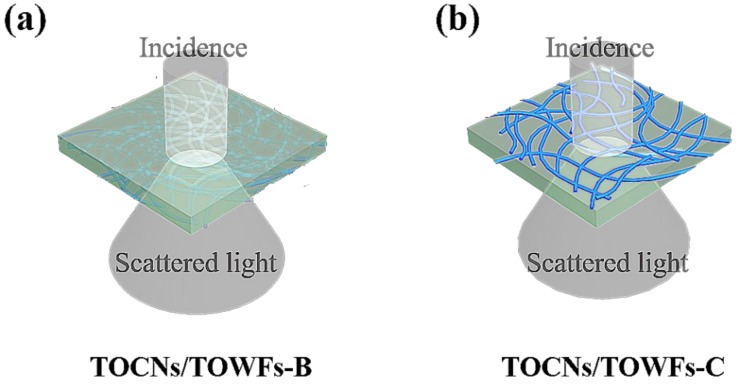
Mechanism of light scattering of (**a**) TOCNs/TOWFs-B films and (**b**) TOCNs/TOWFs-C films.

Figure 3 illustrates the mechanism of light scattering for TOCNs/TOWFs composite films. For the “blending” method (similar to particle-diffusing type), high optical haze was mainly achieved through the serious light scattering of the micro-sized fibers within the transparent films (Figure 3a). For the “coating” method (similar to surface-relief type), the forming of the surface-relief microstructures via the deposition of micro-sized TOWFs induced strong light scattering and endowed high optical haze for the transparent films (Figure 3b).

### 3.2. Surface and Cross-Sectional Morphology

**Figure 4:**
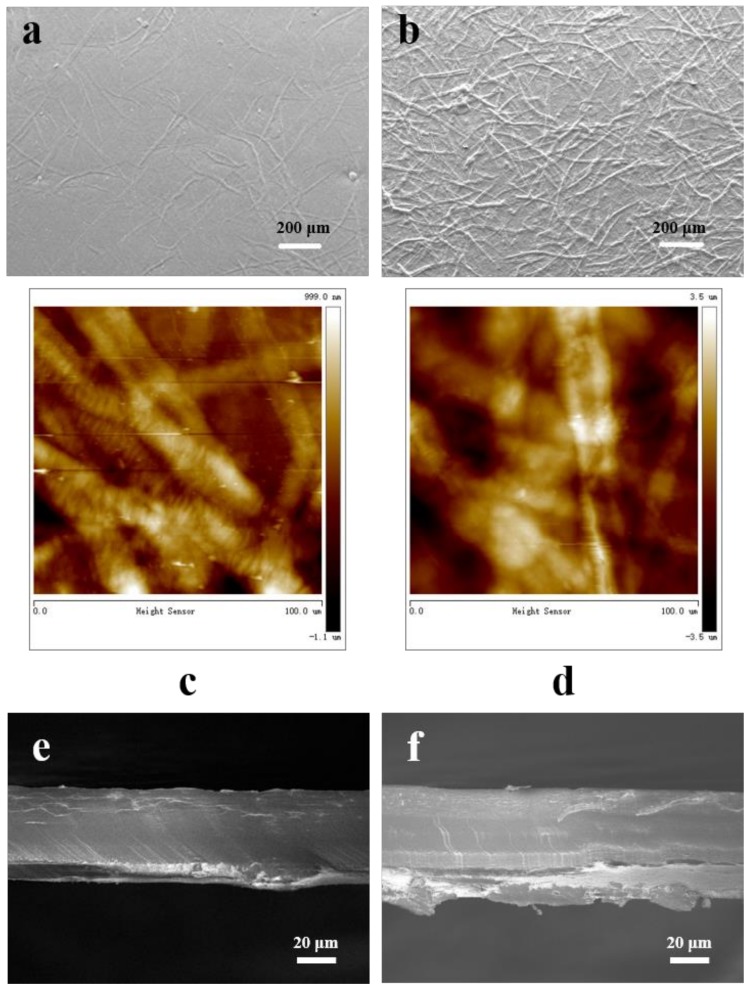
(**a**) FE-SEM image of TOCNs/TOWFs-B films, (**b**) FE-SEM image of TOCNs/TOWFs-C films, (**c**) 3D AFM image of TOCNs/TOWFs-B films surface, (**d**) 3D AFM image of the rough surface of TOCNs/TOWFs-C films, (**e**) cross-sectional structure of TOCNs/TOWFs-B films, and (**f**) cross-sectional structure of TOCNs/TOWFs-C films.

As shown in Figure 4a,b, two types of TOCNs/TOWFs hybrid films had a condensed structure and no obvious holes, which endowed a high total transmittance for these transparent films [26]. For the TOCNs/TOWFs-B films, TOCNs and TOWFs were tightly intertwined together to form a uniform and compact structure. As the top-view SEM images show, only a few micro-sized fibers were observed on the surface of the hybrid films. For the TOCNs/TOWFs-C films, TOWFs were directly coated on the TOCN film surface to form surface-relief morphology. Additionally, a randomly intertwined TOWFs network was observed clearly in the top-view SEM images (Figure 4b). To further investigate the surface roughness, atomic force microscopy (AFM) was used to analyze TOCNs/TOWFs hybrid film surfaces. The particular values are shown in Table 1. The dual sides of the TOCNs/TOWFs-B films had a similar surface roughness of 279 nm (Figure 4c). The TOCN films consisting nano-sized TOCNs, prepared by the solution-casting method, had an extremely smooth surface with RMS of 6.25 nm. Then, the TOWFs were coated on the one side of the TOCN films to form a rough TOWF layer with RMS of 918 nm, and the other one was kept smooth. Hence, the dual sides of the TOCNs/TOWFs-C films had different surface morphologies; one side was smooth, and the other one was rough. The rough surface endowed high optical haze for the transparent films, and simultaneously the smooth surface was suitable for continuous conductive thin layer (~100 nm) depositing. Under the same addition of TOWFs, the hazy film prepared by “coating” method exhibited a rougher surface morphology than the “blending” method. As our previous study showed, the rough surface caused substantial light scattering, which led to a higher optical haze. Hence, the TOCNs/TOWFs-C film had a higher optical haze than TOCNs/TOWFs-B film. As the TOCNs and TOWFs were tightly intertwined with each other, the blade-cut cross-sections were very smooth, and the internal structures could not be clearly observed (Figure 4e,f). The dense structure endowed TOCNs/TOWFs hybrid film’s high light transmittance.

### 3.3. Thermal Property

**Figure 5:**
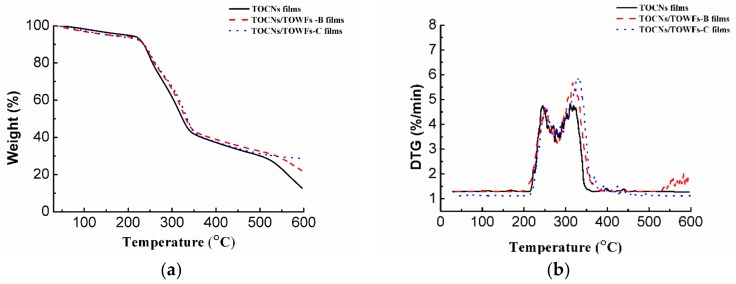
Thermogravimetric analysis (TGA) (**a**) and derivative thermogravimetry (DTG) (**b**) curves of TOCN films, TOCNs/TOWFs-B films, and TOCNs/TOWFs-C films.

TOCN films are more suitable for manufacturing transparent electronic devices than common plastic materials due to their excellent thermal stability and degradation behaviors [27]. Thermal stability is one of the most crucial properties of polymer materials, which can influence the processing and service life performance of these materials [28,29]. Thermogravimetric analysis (TGA) and derivative thermogravimetry (DTG) curves of the transparent films are presented in Figure 5, and the corresponding characteristic data are listed in Table 2. All the film samples had a small weight loss in the low temperature (<100 °C), due to the evaporation of absorbed water. The thermal transition of the films includes the tow phase: (1) the decomposition of carboxyl groups occurred at about 250 °C and (2) the decomposition of cellulose occurred at about 320 °C [30]. Among the three samples, the Stage I maximum thermal degradation temperature (Tmax) of the TOCN films occured at 244 °C with a maximum weight loss rate (WLRmax) of 4.7%/min, and the corresponding data for Stage II are 319 °C and 4.7%/min. The thermal stability of TOCNs/TOWFs-B and TOCNs/TOWFs-C films (“blending” method and “coating” method) is similar to that of TOCN films. TOCNs/TOWFs-B and TOCNs/TOWFs-C films only had a higher char yield than neat TOCN films. Although the thermal stability of the TOCNs/TOWFs hybrid films (degradation at around 250 °C) was lower than the natural cellulose fibers (degradation at around 300 °C [31]), it was still higher than most of the flexible plastics, such as polyethylene terephthalate (PET), polyethylene naphthalate (PEN), and polycarbonate (PC) [32].

**Figure 6:**
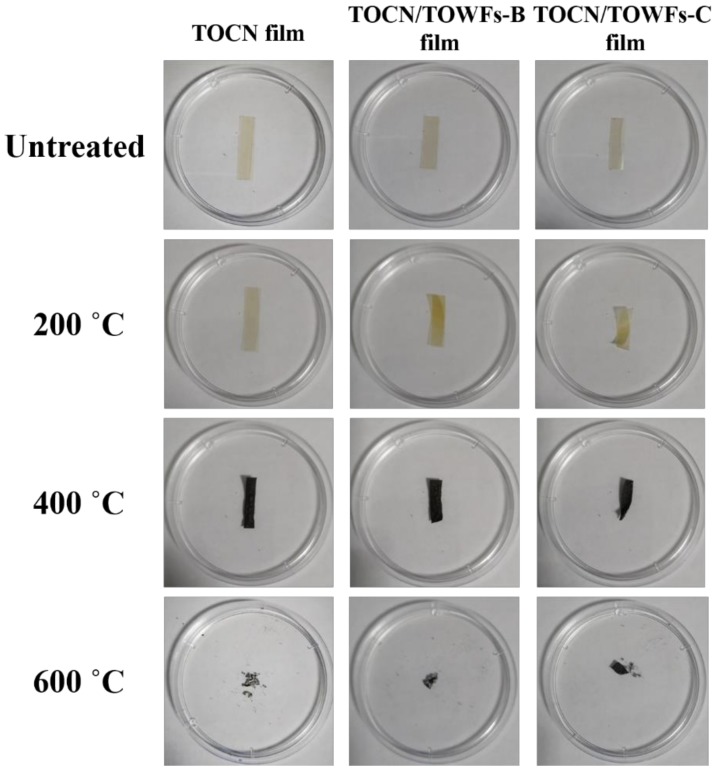
Thermal stability of TOCN films, TOCNs/TOWFs-B films, and TOCNs/TOWFs-C films under air atmosphere.

To further demonstrate the thermal stability of the samples, TOCN films and TOCNs/TOWFs films were tested in a muffle furnace while the morphology of the film samples before and after thermal treatment was observed. The morphology of the film samples is shown in Figure 6. When temperature reached 200 °C, the morphology of the film samples remained unchanged. As the temperature was further increased, the film samples began to carbonize and eventually burned to ash. These results indicated that the hazy and transparent film has an excellent morphology stability under a relatively low temperature (less than 200 °C).

### 3.4. Mechanical Property

The mechanical properties of transparent substrates play a significant role in flexible and transparent devices [33]. A comparative study of the mechanical properties of TOCNs/TOWFs hybrid films prepared by different fabrication methods was carried out. The tensile properties of film samples are listed in Table 3. Mechanical properties relate directly to the single fibril and interfibrillar bonding, which also could be affected by the fabrication processes. The TOCN films, comprised of nano-scale TOCNs, exhibited a remarkably high strength of 92 MPa, high Young’s modulus of 9.81 GPa due to the tightly intertwined nanofibrillar network, and numerous hydrogen bonds [34]. In comparison with TOCNs, TOWFs had a bigger dimension and smaller specific surface area, resulting in a loose stricture for TOCNs/TOWFs hybrid films. Hence, TOCNs/TOWFs-B and TOCNs/TOWFs-C films (“blending” method and “coating” method) showed a lower tensile strength of 75 MPa and 80 MPa, respectively, than neat TOCN films. However, the stiffness of the TOCNs/TOWFs-B and TOCNs/TOWFs-C films was greater than neat TOCN film. In general, the tensile property is still much higher than those of common synthetic polymer films [35].

### 3.5. Resistance to Corrosive Chemicals and Photodegradation Properties

**Figure 7:**
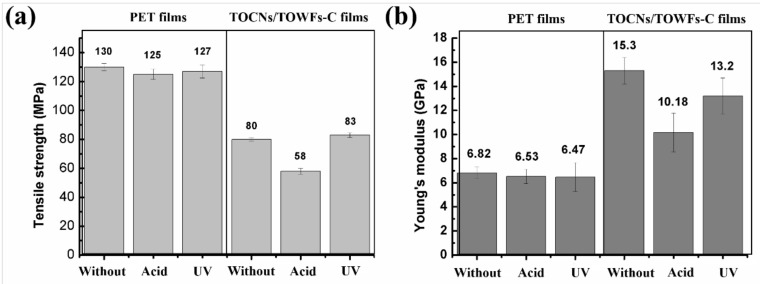
Mechanical properties of the TOCNs/TOWFs-C films and PET films without treatment, with acid or UV radiation treatment, (a) Tensile strength, (b) Young’s modulus.

It is very important to identify the stability of film samples in extreme conditions when they are applied to outdoor displays or solar cells. The corrosion- and photodegradation-resistant properties of the film samples were estimated by measuring the mechanical properties of the film samples after dilute acid or UV radiation treatments. Figure 7 shows the mechanical properties of the film samples after dilute acid (0.1 mol/L, pH = 1.0) or UV radiation treatments for 24 h. In the case of immersion in 0.1 mol/L hydrochloric acid, the tensile strength of TOCNs/TOWFs-C films decreased from 80 MPa to 58 MPa; simultaneously, Young’s modulus decreased from 15.30 GPa to 10.18 GPa. These results indicated that the mechanical property of the TOCNs/TOWFs-C films was reduced by immersion in the acid solution. In the case of UV radiation for 24 h, the mechanical property was kept almost constant. These results indicated that the TOCNs/TOWFs-C films were not acid-resistant but ultraviolet radiation-resistant. As a reference, the corrosion- and photodegradation-resistant properties of PET films were studied. In the case of dilute acid treatment or UV radiation for 24 h, the mechanical properties of the PET films were kept almost constant. Hence, in comparison with traditional PET plastic films, TOCNs/TOWFs-C films are not acid-resistant, which needs to be further improved to meet the requirements of the outdoor application.

## 4. Conclusions

In this study, highly transparent and hazy films (85% total transparency and 62% optical haze at 600 nm wavelength) were prepared by coating micro-sized TEMPO-oxidized fibers onto TOCN film surfaces. The TOCNs/TOWFs-C films had dual sides with different microstructures: one side was smooth (root-mean-square roughness of 6.25 nm) and the other was rough (root-mean-square roughness of 918 nm). The rough surface endowed a high haze for films without seriously decreasing total transmission. The optical haze property of TOCNs/TOWFs-C films was exceptionally good, and much superior to TOCNs/TOWFs-B prepared by blending TOCNs and TOWFs. In addition, the smooth surface of TOCNs/TOWFs-C was more suitable for the conductive thin layer depositing than TOCNs/TOWFs-B films. Both TOCNs/TOWFs-B and TOCNs/TOWFs-C films exhibited good thermal stability and mechanical properties. The resulting films also showed excellent ultraviolet radiation resistance but not acid resistance. Our work provided insight into the preparation of high performance transparent and hazy films for the fabrication of green optoelectronics. 

## Figures and Tables

**Table 1:** Thickness, surface roughness, and optical properties of TOCN films, TOCNs/TOWFs-B films, and TOCNs/TOWFs-C films.

Sample	Thickness (μm)	Upper Surface Roughness (nm)	Bottom Surface Roughness (nm)	Transmittance (%)	Haze (%)
TOCN films	34.6	6.25	6.25	89	3.8
TOCNs/TOWFs-B films	69.2	279	279	83	50
TOCNs/TOWFs-C films	80.5	918	6.25	85	62

**Table 2:** TGA and DTG data measured at a heating rate of 10 °C/min.

Sample	Stage I		Stage II		CY (%)
	Tmax (°C)	WLRmax (%/min)	Tmax (°C)	WLRmax (%/min)	
TOCN films	244	4.7	319	4.7	12.7
TOCNs/TOWFs-B films	250	4.8	318	5.7	22.0
TOCNs/TOWFs-C films	250	4.6	327	5.9	28.7

**Table 3:** Tensile properties of the polyethylene terephthalate (PET) films, TOCN films, TOCNs/TOWFs-B films, and TOCNs/TOWFs-C films.

Sample	Tensile Strength (MPa)	Young’s Modulus (GPa)	Strain at Break (%)
TOCN films	92 ± 6.5	9.81 ± 1.3	0.98 ± 0.2
TOCNs/TOWFs-B films	75 ± 3.5	10.49 ± 2.1	0.75 ± 0.1
TOCNs/TOWFs-C films	80 ± 0.1	15.30 ± 1.1	0.78 ± 0.2

## References

[1] Chung H.H., Sun L. (2012). Contrast-ratio analysis of sunlight-readable color LCDs for outdoor applications. J. Soc. Inf. Disp..

[2] Brongersma M.L., Cui Y., Fan S. (2014). Light management for photovoltaics using high-index nanostructures. Nat. Mater..

[3] Ko M., Baek S.H., Song B., Kang J.W., Cho C.H. (2016). Periodically diameter-modulated semiconductor nanowires for enhanced optical absorption. Adv. Mater..

[4] Huang T.C., Ciou J.R., Huang P.H., Hsieh K.H., Yang S.Y. (2008). Fast fabrication of integrated surface-relief and particle-diffusing plastic diffuser by use of a hybrid extrusion roller embossing process. Opt. Express.

[5] Liu S.J., Huang Y.C. (2009). Manufacture of dual-side surface-relief diffusers with various cross angles using ultrasonic embossing technique. Opt. Express.

[6] Hassinen T., Eiroma K., Mäkelä T., Ermolov V. (2015). Printed pressure sensor matrix with organic field-effect transistors. Sen. Actuator A-Phys..

[7] Koga H., Nogi M., Komoda N., Nge T.T., Sugahara T., Suganuma K. (2014). Uniformly connected conductive networks on cellulose nanofiber paper for transparent paper electronics. NPG. Asia. Mater..

[8] Shih T.K., Chen C.F., Ho J.R., Chuang F.T. (2006). Fabrication of PDMS (polydimethylsiloxane) microlens and diffuser using replica molding. Microelectron. Eng..

[9] Chong Y.P., Kim J.M., Sun I.K., Yong N.H., Choi Y.S. (2003). Holographic diffuser by use of a silver halide sensitized gelatin process. Appl. Opt..

[10] Sakai D., Harada K., Kamemaru S.I., El-Morsy M.A., Itoh M., Yatagai T. (2005). Direct fabrication of surface relief holographic diffusers in azobenzene polymer films. Opt. Rev..

[11] Chang S.I., Yoon J.B., Kim H., Kim J.J., Lee B.K., Shin D.H. (2006). Microlens array diffuser for a light-emitting diode backlight system. Opt. Lett..

[12] Méndez E.R., García-Guerrero E.E., Escamilla H.M., Maradudin A.A., Leskova T.A., Shchegrov A.V. (2001). Photofabrication of random achromatic optical diffusers for uniform illumination. Appl. Opt..

[13] Parikka M., Kaikuranta T., Laakkonen P., Lautanen J., Tervo J., Honkanen M., Kuittinen M., Turunen J. (2001). Deterministic diffractive diffusers for displays. Appl. Opt..

[14] Fang Z., Zhu H., Preston C., Han X., Li Y., Lee S., Chai X., Chen G., Hu L. (2013). Highly transparent and writable wood all-cellulose hybrid nanostructured paper. J. Mater. Chem. C..

[15] Zhu H., Fang Z., Zhu W., Dai J., Yao Y., Fei S., Preston C., Wu W., Peng P., Jang N. (2015). Extreme light management in mesoporous wood cellulose paper for optoelectronics. ACS Nano.

[16] Hsieh M.C., Koga H., Suganuma K., Nogi M. (2017). Hazy transparent cellulose nanopaper. Sci. Rep-UK.

[17] Yang W., Bian H., Jiao L., Wu W., Deng Y., Dai H. (2017). High wet-strength, thermally stable and transparent TEMPO-oxidized cellulose nanofibril film via cross-linking with poly-amide epichlorohydrin resin. RSC Adv..

[18] Saito T., Nishiyama Y., Putaux J.L., Vignon M., Isogai A. (2006). Homogeneous suspensions of individualized microfibrils from TEMPO-catalyzed oxidation of native cellulose. Biomacromolecules.

[19] Plastics D.O. (2012). Standard Test Method for Haze and Luminous Transmittance of Transparent Plastics.

[20] Hu L., Zheng G., Yao J., Liu N., Weil B., Eskilsson M., Karabulut E., Ruan Z., Fan S., Bloking J.T. (2013). Transparent and conductive paper from nanocellulose fibers. Energ. Environ. Sci..

[21] Baumgartner M., Coppola M.E., Sariciftci N.S., Glowacki E.D., Bauer S., Irimia-Vladu M. (2017). Emerging “green” Materials and technologies for electronics. Green Materials for Electronics.

[22] Ha D., Fang Z., Zhitenev N.B. (2018). Paper in electronic and optoelectronic devices. Adv. Electron. Mater..

[23] Zhou P., Zhu P., Chen G., Liu Y., Kuang Y., Liu Y., Fang Z. (2018). A study on the transmission haze and mechanical properties of highly transparent paper with different fiber species. Cellulose.

[24] Yang W., Jiao L., Min D., Liu Z., Dai H. (2017). Effects of preparation approaches on optical properties of self-assembled cellulose nanopapers. RSC Adv..

[25] Yang W., Jiao L., Liu W., Deng Y., Dai H. (2018). Morphology control for tunable optical properties of cellulose nanofibrils films. Cellulose.

[26] Yan Q., Sabo R., Wu Y., Zhu J.Y., Cai Z. (2015). Self-assembled optically transparent cellulose nanofibril films: effect of nanofibril morphology and drying procedure. Cellulose.

[27] Yu H., Qin Z., Liang B., Liu N., Zhou Z., Chen L. (2013). Facile extraction of thermally stable cellulose nanocrystals with a high yield of 93% through hydrochloric acid hydrolysis under hydrothermal conditions. J. Mater. Chem. A.

[28] Zhang Y., Heo Y., Son Y. (2018). Recent advanced thermal interfacial materials: A review of conducting mechanisms and parameters of carbon materials. Carbon.

[29] Zhang Y., Choi J., Park S. (2018). Interlayer polymerization in amine-terminated macromolecular chain-grafted expanded graphite for fabricating highly thermal conductive and physically strong thermoset composites for thermal management applications. Compos. Part A Appl. S..

[30] Sun X., Wu Q., Ren S., Lei T. (2015). Comparison of highly transparent all-cellulose nanopaper prepared using sulfuric acid and TEMPO-mediated oxidation methods. Cellulose.

[31] Li Y., Zhu H., Xu M., Zhuang Z., Xu M., Dai H. (2014). High yield preparation method of thermally stable cellulose nanofibers. Bioresources.

[32] Mecking S. (2010). Nature or petrochemistry-biologically degradable materials. Angew. Chem. Int. Edit..

[33] Zhu H., Fang Z., Preston C., Li Y., Hu L. (2013). Transparent paper: Fabrications, properties, and device applications. Energ. Environ. Sci..

[34] Benítez A.J., Torresrendon J., Poutanen M., Walther A. (2013). Humidity and multiscale structure govern mechanical properties and deformation modes in films of native cellulose nanofibrils. Biomacromolecules.

[35] Siró I., Plackett D., Siro I., Plackett D. (2010). Microfibrillated cellulose and new nanocomposite materials: A review. Cellulose.

